# Epithelial-to-mesenchymal transition leads to loss of EpCAM and different physical properties in circulating tumor cells from metastatic breast cancer

**DOI:** 10.18632/oncotarget.8250

**Published:** 2016-03-22

**Authors:** Kyung-A Hyun, Gi-Bang Koo, Hyunju Han, Joohyuk Sohn, Wonshik Choi, Seung-Il Kim, Hyo-Il Jung, You-Sun Kim

**Affiliations:** ^1^ School of Mechanical Engineering, Yonsei University, Seoul, Korea; ^2^ Department of Biochemistry, Ajou University School of Medicine, Suwon, Korea; ^3^ Department of Biomedical Sciences, Graduate School, Ajou University, Suwon, Korea; ^4^ Institute for Cancer Research, Yonsei University College of Medicine, Seoul, Korea; ^5^ Department of Medical Oncology, Yonsei University College of Medicine, Seoul, Korea; ^6^ Department of Physics, Korea University, Seoul, Korea; ^7^ Department of Surgery, Yonsei University College of Medicine, Seoul, Korea

**Keywords:** epithelial cell adhesion molecule (EpCAM), EpCAM-negative, EMT-induced breast cancer cell, circulating tumor cells (CTCs), label-free separation

## Abstract

The dissemination of circulating tumor cells (CTCs) requires the Epithelial-to-Mesenchymal transition (EMT), in which cells lose their epithelial characteristics and acquire more mesenchymal-like phenotypes. Current isolation of CTCs relies on affinity-based approaches reliant on the expression of Epithelial Cell Adhesion Molecule (EpCAM). Here we show EMT-induced breast cancer cells maintained in prolonged mammosphere culture conditions possess increased EMT markers and cancer stem cell markers, as well as reduced cell mass and size by quantitative phase microscopy; however, EpCAM expression is dramatically decreased in these cells. Moreover, CTCs isolated from breast cancer patients using a label-free microfluidic flow fractionation device had differing expression patterns of EpCAM, indicating that affinity approaches reliant on EpCAM expression may underestimate CTC number and potentially miss critical subpopulations. Further characterization of CTCs, including low-EpCAM populations, using this technology may improve detection techniques and cancer diagnosis, ultimately improving cancer treatment.

## INTRODUCTION

Circulating tumor cells (CTCs), located in the peripheral blood of cancer patients, are highly correlated with the invasive behavior of some types of cancer. Therefore, the precise detection and isolation of CTCs may be a powerful tool in cancer prognosis, diagnosis of minimal residual disease, assessment of tumor sensitivity to anticancer drugs, and personalization of anticancer therapy. In recent years, several studies have reported on the correlation between the presence of CTCs and clinical outcomes, such as overall survival (OS) and progression-free survival (PFS), in metastatic breast cancer patients [1]. There has been major progress in detecting CTCs in peripheral blood over the last decade due to the development of CTC-enrichment technologies, based on expression of the Epithelial Cell Adhesion Molecule (EpCAM) [2, 3]. However, epithelial tumor cells often undergo epithelial-mesenchymal transition (EMT), enabling them to invade blood vessels, survive in the blood stream and invade other organs [4], and in the process, CTCs undergo phenotypic changes, such as loss of epithelial marker expression, and acquiring a stem cell-like phenotype [5, 6]. Thus, we hypothesize that some CTCs may lose expression of EpCAM. Because CTCs are rare in peripheral blood, missing EpCAM-negative CTCs in a given patient might be the equivalent of missing all CTCs in that patient, thus exposing a problematic limitation of CTC-enrichment technologies that rely on affinity-based capture exploiting the anti-EpCAM antibody [7–9]. Standardized detection and isolation methodologies, as well as single cell omics technologies are therefore likely to be at the forefront of the CTC field [10].

Label-free separation approaches exploit the biophysical properties of target cells, such as their size, shape, density, and deformability. The advantages of these approaches are that they enable the collection of intact heterogeneous CTCs, regardless of their surface marker expression level, at high throughput and low cost. We recently developed a parallel multi-orifice flow fractionation (p-MOFF) chip for high-throughput size-based CTC separation [11]. Within each of the MOFF channels, leukocytes, which are smaller than CTCs, are split laterally into two positions, because leukocytes experience less inertial lift force from the series of contraction/expansion channels. CTCs are focused at the center of the channel due to the wall effect-induced lift force. Consequently, at the end of the channels, the leukocytes are released to the outlets for waste, and the CTCs are collected in the appropriate outlet.

To investigate EpCAM expression heterogeneity in circulating tumor cells, we designed a model system for EMT-induced breast cancer cells. Using this model system, we analyzed the physical and molecular characters of EMT-induced breast cancer cells, which have low levels of EpCAM expression. Using our p-MOFF system, we demonstrated efficient isolation of CTCs regardless of heterogeneous EpCAM expression in breast cancer patient blood samples. We believe that this method will improve our understanding of CTC biology and provide a substantive understanding of the molecular nature of CTCs in relation to clinical applications.

## RESULTS

### EMT phenotype of cancer cells can have different physical properties

**Figure 1:**
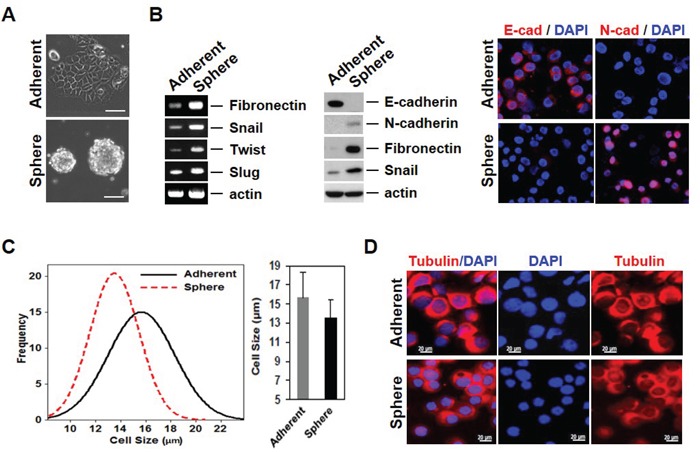
Mammosphere cultured MCF-7 cells acquire EMT phenotypes with variability in properties **A.** Mammosphere culture of MCF-7 cells. MCF-7 cells were cultured in mammosphere culture media and representative images were taken by a phase-contrast microscope. **B.** mRNA and protein levels of EMT-related genes were increased in sphere cells. RT-PCR was performed to see the gene expression in the RNA sample. Cell lysates were also confirmed by Western blot. The E/N-cadherin switch in sphere cultured cells (left). Protein levels of EMT-related genes were analyzed by immunofluorescence staining (right). **C.** Measure of cell size using the Olympus IX81-ZDC inverted microscope for MCF-7 and mammosphere-cultured MCF-7 cells. **D.** Images of cell morphology in adherent and sphere cells. Cells were cytospun onto glass slides for immunofluorescence staining with anti-tubulin antibody. Representative merged images were derived from tubulin/DAPI. Scale bars representing 20 μm were added from an image taken at identical magnification and resolution.

**Figure 2:**
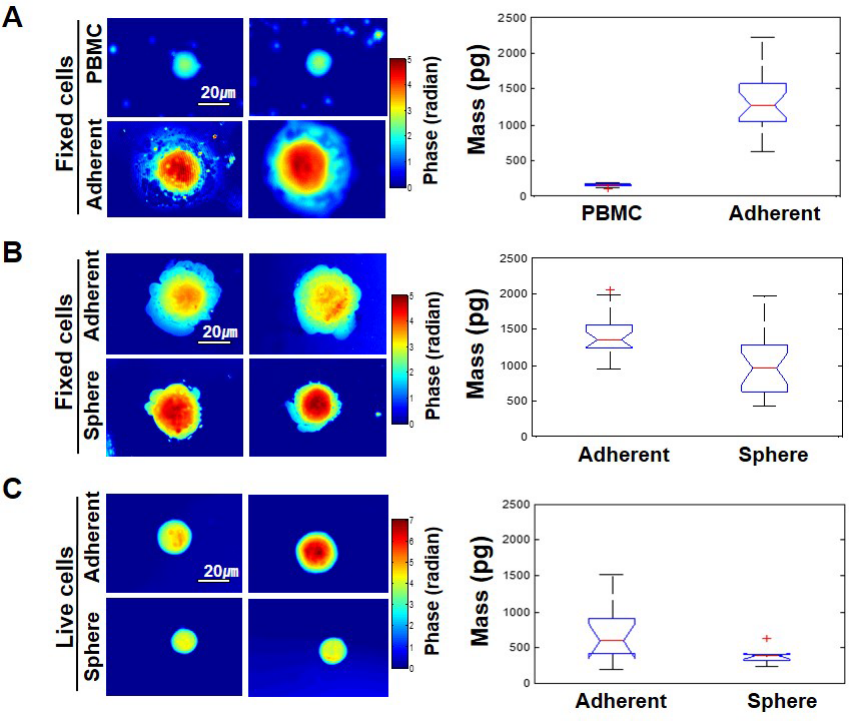
The EMT phenotype in cells has different physical properties **A.** Left-hand panel: representative phase maps of PBMC and MCF-7 cells, both patched on the coverslips. Color bar indicates phase in radians. Right-hand panel: distribution of cell mass for 25 cells per each group (p-value = 5.4 x10^−8^). **B.** Comparison between adherent MCF-7 cells and mammosphere-cultured MCF-7 cells, both patched on the coverslips. Twenty cells were analyzed per each group and the p-value was 0.0003. **C.** The same as (B) but cells were not fixed and were floating free in the media for live cells. Ten cells were analyzed per each group and the p-value was 0.031.

Most currently used assays for detecting CTCs are based on EpCAM expression. However, some cancer cells have little or no EpCAM expression. The heterogenous expression of EpCAM in cancer cells may be related to the EMT process [6]. For instance, we have previously reported that EpCAM-negative breast cancer cells express high amounts of EMT-related genes [10, 12]. Mammosphere culture has been utilized to enrich for both normal and cancer populations of stem cells (CSCs), as well as to initiate EMT [14, 17, 18]. We thus established a cell model system for mammosphere-induced EMT. In this model system, MCF-7 cells (Adherent) showed tightly aggregated spheroids (Sphere); sphere cells expressed various EMT-related genes such as fibronectin, snail1, twist, and slug (Figure 1A and B). A reduction in cell-cell adhesion was observed, which was associated with a marked decrease in E-cadherin expression and an increase in N-cadherin in sphere cells as measured by Western blotting and immunofluorescence staining. Marrinucci *et al*. reported CTCs to be a heterogeneous population, within and across patients, as CTCs had various shapes, sizes, and cytokeratin expression [15]. Since our data suggest that serial mammosphere culture of cancer cells induces the EMT process, and these cells show EMT-related changes in gene expression, we further explored the physical properties of EMT-induced cancer cells. EMT-induced sphere cells became smaller than adherent cells (Figure 1C and 1D). Next, we used a quantitative phase microscope [16] to measure the non-aqueous mass of peripheral blood mononuclear cells (PBMCs) in comparison to the adherent and sphere cells when patched on coverslips. The adherent MCF-7 cells were at least 7 times larger in mass than the PBMCs (Figure 2A). Sphere cells were smaller than adherent cells in size, and there was a statistically significant reduction in mass after mammosphere culture (Figure 2B and 2C). We further confirmed these tendencies in live cells.

### Cancer cells with an EMT phenotype display reduced EpCAM expression

**Figure 3:**
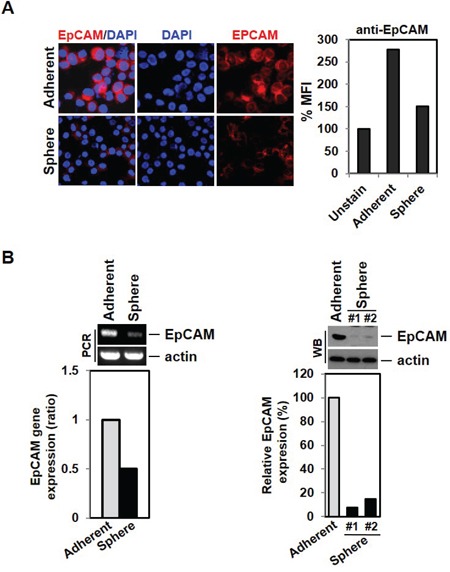
Down-regulation of EpCAM expression by EMT induction **A.** Low expression of EpCAM cell surface marker expression in mammosphere-cultured cells. MCF-7 and sphere cultured cells were stained with EpCAM antibody and analyzed by fluorescence microscope and FACS analysis. **B.** The EMT phenotype leads to decreased expression levels of the EpCAM gene and protein. RNA from MCF-7 cells and cultured sphere cells were isolated and analyzed by RT-PCR. Cell lysates were also analyzed by Western blot.

To test the possibility that MCF-7 cells have reduced EpCAM expression in response to EMT induction, we stained cells with EpCAM antibody. Indeed, mammosphere-cultured cells show less EpCAM expression than adherent cells when visualized under a fluorescence microscope or measured by FACS (Figure 3A). Consistent with microarray data we had previously obtained (data not shown), RT-PCR indicated that EpCAM mRNA was substantially reduced in MCF-7 cells sphere cells (as normalized to actin levels) (Figure 3B left panel). EpCAM protein expression was also reduced in the mammosphere-cultured MCF-7 as measured by western blotting (Figure 3B, right panel). These data indicate that EMT induction may result in decreased EpCAM expression level, thereby limiting EpCAM-based detection for the identification of CTCs. It suggests that relying on EpCAM expression for detection of CTCs could result in a failure to detect many cancer cells that have undergone EMT in the bloodstream in patients.

### The EMT phenotype is associated with increased expression of cancer stem cell markers and resistance to chemotherapeutic agents

**Figure 4:**
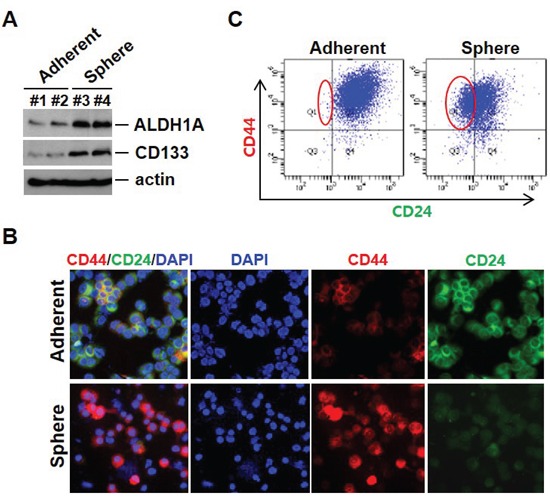
Cancer stem-like cells can arise as a result of EMT **A.** Cancer stem cell–related marker expression in mammosphere cultured cells. Cell lysates from adherent cells and mammosphere cultured cells were analyzed by Western blot with indicated antibodies. **B.** Mammosphere cultured cells show CD44 high/CD24 low expression at the cell surface. Cells were cytospun onto glass slides for CD44 and CD24 antibody staining. Representative images were taken by fluorescence microscopy. **C.** Analysis using flow cytometry further revealed that sphere cells exhibited an increase in CD44 high/CD24 low populations.

EMT has been reported to result in cells with “stem cell-like” properties that are more resistant to chemotherapeutic agents [19]. ALDH1A1 and CD133 have been reported as cancer stem cell markers and overexpression of EMT markers on CTCs is often accompanied by ALDH1 expression in breast cancer at all stages of the disease. As shown in Figure 4A, sphere cells expressed high levels of CD133 and ALDH1A1; these cells additionally showed increased CD44 and decreased CD24 by immunofluorescence staining and FACS analysis, a phenotype associated with potential breast CSCs (Figure 4B and 4C). ALDH1A1-positive CTCs were also identified from the blood of metastatic breast cancer patients (data not shown).

**Figure 5:**
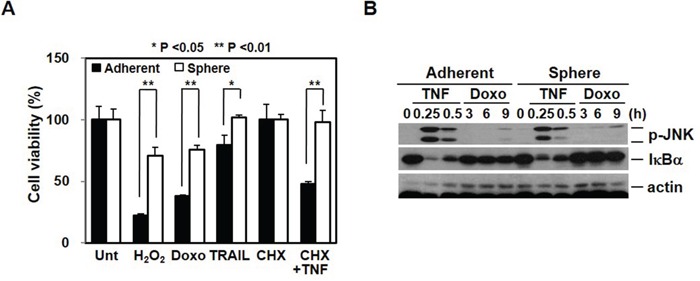
Chemoresistance is associated with cancer stem cell-like properties and EMT **A.** Cancer stem cell-like cells are more resistant to death signals. Adherent and sphere cells were treated with H2O2 (1 mM), doxorubicin (5 μM), TRAIL (100 ng/mL), and cycloheximide (CHX, 5 μg/mL) plus TNFα (30 ng/mL) for 24 hrs. Cell viability was analyzed by MTT assay. **B.** Extrinsic and intrinsic signaling pathways were not difference after mammosphere cultured cells. Adherent cells and mammosphere cultured cells were treated with TNFα (30 ng/mL) or doxorubicin (2.5 μM) for the indicated time points and cell lysates were analyzed by Western blot.

Consistent with the evidence that breast CSCs have a high resistance to chemotherapy [20], our sphere cells, which have a high level of cancer stem cell properties, are more resistant than adherent cells to various death signals (Figure 5A). However, although mammosphere-cultured cells show different gene expression patterns and have differential responses to death stimuli, the adherent and sphere cells showed similar signaling events with respect to outside signals. Cells were treated with TNF to examine cell surface receptor response and with doxorubicin to examine the intrinsic response. As shown in Figure 5B, in both groups of cells, a similar pattern of JNK phosphorylation and IκBα degradation was shown.

### Operating procedure for characterization of CTCs

**Figure 6:**
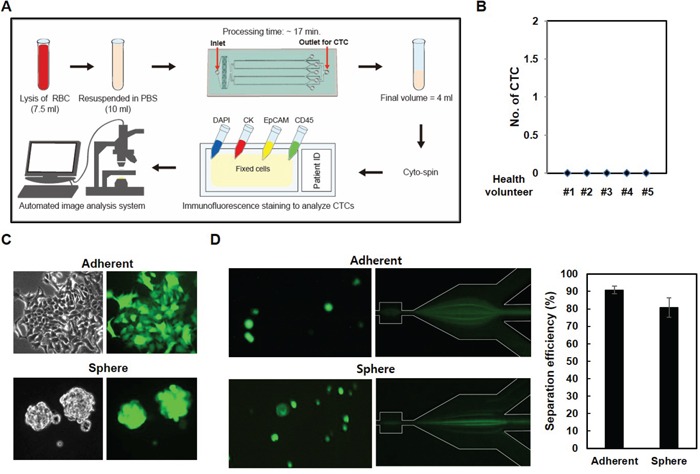
CTC isolation using a p-MOFF chip **A.** Schematic drawing of the experimental setup of the p-MOFF chip and the CTC analysis procedures. **B.** Blood samples from healthy volunteers were injected into the p-MOFF chip and isolated cells were stained with DAPI, anti-CD45, anti-EpCAM, and anti-cytokeratin antibodies. **C.** Establishment of GFP-labeled adherent and sphere cells. The cells were infected with GFP-lentiviral construct to establish the stable GFP-expressing cells. **D.** Cells from (C) injected into the p-MOFF chip (left). The separation efficiency of adherent and sphere MCF-7 cells using the p-MOFF chip was 90.78% for adherent MCF-7 cells and 80.81% for sphere MCF-7 cells (right).

Since our data suggested that CTCs display heterogenic gene expression profiles, especially with regard to EpCAM and to some cell surface proteins, we isolated CTCs using a parallel multi-orifice flow fractionation (p-MOFF) chip, which allows continuous isolation of EpCAM-positive and EpCAM-negative CTCs from the whole blood of breast cancer patients. The operating procedure for isolation and characterization of CTCs is summarized in Figure 6A. First, the lysis buffer for red blood cells (Qiagen, Chatsworth, CA) was added to 7.5 mL whole blood sample in a 1:10 v/v ratio and incubated for 10 minutes at room temperature. After removing the RBCs, the remaining pellet was re-suspended in 10 ml PBS containing 3% BSA and then sample was injected into the inlet of a p-MOFF chip with injection flow rate 600 μL/min and the CTCs were collected into the syringe which connected to the CTC outlet of the chip with a withdrawal flow rate of 240 μL/min as maintained by syringe pumps (KDS210, KD Scientific Inc., MA, USA). The cells collected from the outlet for CTC were fixed on a glass slide using cytospin, and the cells subsequently were stained for DAPI, cytokeratin7/8, EpCAM, and CD45 to identify nuclei, CTCs, epithelial cells, and leukocytes, respectively. Finally, the slides were fitted on an automated image analysis system, and photographed by a moving stage linked with an image-acquisition system, which was programmed using MATLAB® (MathWorks, Massachusetts, USA).

To define the positive and negative immune-phenotype for each cell, the leukocytes from a healthy donor and three breast cancer cell lines (MCF-7, SK-BR-3 and MDA-MB-231) were individually stained (Figure S1). The intensity of immunofluorescence signals was characterized by the image-processing program, Image J (NIH, Maryland, USA). The highest cytokeratin intensity of leukocytes was used as the CTC cut-off value. Similarly, the highest CD45 intensity of the three breast cancer cell lines was used for the leukocyte cut-off value (Figure S2 (a)). The EpCAM intensity was also analyzed in breast cancer cell lines (MCF-7 (EpCAM+) and MDA-MB-231 (EpCAM-)) (Figure S2 (b)). Based on these cut-off values, CTCs were identified as positive for DAPI and cytokeratin, and negative for CD45. Cell size and shape were also considered in the CTC characterization. Importantly, five healthy volunteer samples were run through our device before applying to patient samples, and CTCs were not detected (Figure 6B), consistent with our previous report in which CTCs were not detected in any healthy sample [11]. We also tested EMT-induced sphere cells, which show different patterns of EpCAM expression and different physical properties. EMT-induced sphere cells were infected using the GFP-lenti expression system (Figure 6C) and those cells were applied to the p-MOFF channel. Before being injected into the device, cells sticking together were dispersed in PBS containing 3% BSA. Figure 6D shows the behaviors of adherent and spherical MCF-7 cells in the separation region after flowing through the p-MOFF channel. A few spherical MCF-7 cells moved toward the outside of the channel, however, the sphere MCF-7 cells are about 2 μm smaller than adherent MCF-7 cells, so almost all sphere cells followed the focusing flow into the center of the channel. Using the p-MOFF chip, we separated 90.78±2.21 and 80.81±5.49% of adherent and sphere MCF-7 cells, respectively, indicating that our device enables the isolation and detection of CTCs that exhibit heterogeneous expression of EpCAM (Figure 6D right).

### Isolation of CTCs from metastatic breast cancer patients

**Figure 7:**
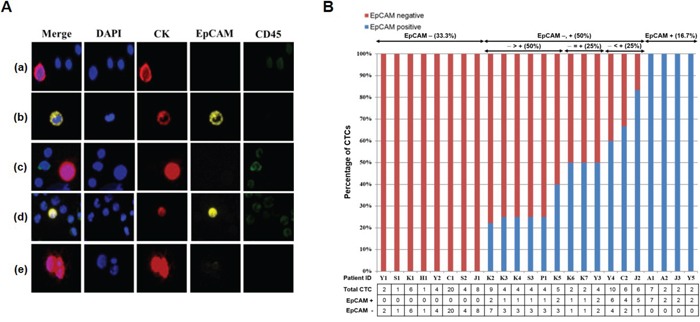
Isolation of CTCs from metastatic breast cancer patients using the p-MOFF chip **A.** Fluorescent images of cells isolated from metastatic breast cancer patients using the p-MOFF chip. There are (a) EpCAM-negative CTCs isolated from a patient who had only EpCAM-negative CTCs and **(b)** EpCAM-positive CTCs isolated from a patient who had only EpCAM-positive CTCs. **(c, d)** Both EpCAM-negative and EpCAM-positive CTCs are seen in patient ID K3. **(e)** A CTC cluster isolated from a patient who had only EpCAM-negative CTCs. **B.** The percentages of EpCAM-negative and EpCAM-positive CTCs isolated from individual patients. There were 33.3% of patients with only EpCAM-negative CTCs and 16.7% of patients with only EpCAM-positive CTCs. Even the percentage of EpCAM-negative CTCs was greater than or equal to that of EpCAM-positive CTCs in patients who had both EpCAM-negative and EpCAM-positive CTCs. The number of heterogeneous CTCs is listed below.

When the blood samples of 32 breast cancer patients were run through our device, one to 20 CTCs were identified in 24 of 32 patients (75%). Figure 7A shows the fluorescent images of cells isolated from metastatic breast cancer patients. Because the label-free p-MOFF chip does not use an EpCAM marker to capture the CTCs, but specifically collects CTCs based on their size, it can separate and collect EpCAM-negative and EpCAM-positive CTCs (Figure 7A (a, b)). We were able, in fact, to identify patients who had both EpCAM-negative and EpCAM-positive CTCs (patient ID K3) Figure 7A (c, d). We also found clustered EpCAM-negative CTCs in patients who had only EpCAM-negative CTCs (Figure 7A (e)). Figure 7B shows the number of heterogeneous CTCs isolated from individual patients. Importantly, only four (16.7%) of the 24 patients had only EpCAM-positive CTCs. This result shows that current EpCAM-based isolation approaches are limited in their ability to detect CTCs in 83.3% of patients (33.3% of patients who had only EpCAM-negative CTCs, 50% of patients who had both EpCAM-negative and EpCAM-positive CTCs) (Figure 7B). EpCAM-negative CTCs were a larger percentage of overall CTCs, even among the patients who had both EpCAM-negative and EpCAM-positive CTCs, as shown in Figure 7B. Thus, isolation of CTCs based on EpCAM alone is insufficient to recover all extant CTCs from patients, and potential biomarkers are needed to improve detection. Our label-free microfluidic flow fractionation device should help molecular characterization of heterogeneous CTCs.

We classified patients into four groups: EpCAM positive, EpCAM negative, EpCAM positive proportion, and EpCAM negative proportion. We then analyzed the association between EpCAM expression on CTCs and characteristics of the patients' cancers (Table 1). There was not significant association in overall parameters. Although the EpCAM positive group tended to have an ER (estrogen receptor) negative status in comparison to EpCAM negative group, it is possible that this association is a random occurrence due to the small number of patients in the cohort.

**Table 1:** Association between EpCAM expression on CTC and clinicopathological parameters

Characteristic		EpCAM				p-value
		Positive(n=4)	Negative(n=8)	Positive proportion (n=6)	Negative proportion (n=6)	
Age		52.25±10.04	45.25±9.16	50.50±13.14	50.33±10.36	0.673
BMI		22.16±1.86	23.68±5.66	23.13±2.99	22.48±3.64	0.923
T stage	Stage 1	2 (50%)	1 (12.5%)	1 (16.7%)	0	0.114
	Stage 2	1( 25%)	3 (37.5%)	4 (66.7%)	5 (83.3%)	
	Stage 3	1 (25%)	0	0	0	
	Stage 4	0	4 (50%)	1 (16.7%)	1 (16.7%)	
N stage	Stage 0	0	0	2 (33.3%)	1 (16.7%)	0.179
	Stage 1	3 (75%)	5 (62.5%)	1 (16.7%)	4 (66.7%)	
	Stage 2	1 (25%)	3 (37.5%)	1 (16.7%)	1 (16.7%)	
	Stage 3	0	0	2 (33.2%)	0	
M stage	Stage 0	4 (100%)	6 (75%)	5 (83.3%)	4 (66.7%)	0.622
	Stage 1	0	2 (25%)	1 (16.7%)	2 (33%)	
TNM stage	Stage 1	0	0	1 (16.7)	0	0.734
	Stage 2	3 (75%)	3 (37.5%)	2 (33.3%)	3 (50%)	
	Stage 3	1 (25%)	3 (37.5%)	2 (33.3%)	1 (16.7%)	
	Stage 4	0	2 (25%)	1 (16.7%)	2 (33.3%)	
ER	Negative	3 (75%)	1 (12.5%)	2 (33.3%)	3 (50%)	0.173
	Positive	1 (25%)	7 (87.5%)	4 (66.7%)	3 (50%)	
PR	Negative	3 (75%)	2 (25%)	5 (83.3%)	3 (50%)	0.136
	Positive	1 (25%)	6 (75%)	1 (16.7%)	3 (50%)	
HER2	Negative	0	6 (75%)	5 (83.3%)	5 (83.3%)	0.102
	Positive	3 (75%)	1 (12.5%)	1 (16.7%)	1 (16.7%)	
	Equivocal	1 (25%)	1 (12.5%)	0	0	

Abbreviations: T stage (Tumor stage), N stage (Lymph node involvement stage), M stage (Metastasis stage), ER (Estrogen receptor), PR (Progesterone receptor), HER2 (Human epidermal growth factor receptor 2)

## DISCUSSION

Several CTC isolation technologies exist, including nucleic acid-based detection, capture using antibodies against cell surface antigens (micropost and magnetic beads), flow cytometry and detection based on physical properties such as differences in size, density and charge [10]. Despite all these methods, CTC isolation and detection still suffers many technical limitations. To date, the most successful approaches have made use of the fact that epithelial cells commonly express the cell adhesion protein EpCAM. The FDA-approved CellSearch (Veridex TM, Raritan, NJ) platform uses immunomagnetic beads coated with antibodies against EpCAM to enrich for EpCAM-expressing cancer cells. EpCAM-based antibody-mediated capture of CTCs is somewhat effective, but only selects for cells expressing this epithelial marker, which, as we have shown here, decreases during the EMT process [21, 22]. The role of EMT in generating CTCs at the primary tumor site has been suggested, so it would seem reasonable to explain the heterogeneity of EpCAM expression. Breast cancer cell lines with low EpCAM expression were primarily identified in a subset of the basal-like subtype and the possible association between EMT and basal-like phenotype was suggested by Sarrio et al. [23]. In the present study, we provide additional evidence that decreased EpCAM expression is correlated with expression of EMT markers and cancer stem cell markers. Based on these finding, cancer stem cell markers and EMT markers could be useful for capturing CTCs from EpCAM-low expressing breast cancer cells.

However, the current biomarkers do not fully cover the heterogeneous population of CTCs and there is still a need to improve the isolation/detection of CTCs that vary in their characteristics. To achieve this, we designed a label-free microfluidic flow fractionation p-MOFF device to isolate EpCAM-negative CTCs. The advantage of this label-free separation approach is that it uses physical properties of CTCs such as size, shape and density, enabling the high-throughput collection of intact heterogeneous CTCs regardless of surface marker expression level. We demonstrated that the p-MOFF system could isolate all CTCs in a breast cancer patient blood sample. Importantly, only 4 of 24 breast cancer patients (16.6%) had only EpCAM-positive CTCs. These results provide evidence that a large number of EpCAM-negative CTCs exist in the blood of metastatic breast cancer patients. Therefore, the ability to collect a large number of intact and heterogeneous CTCs using this label-free microfluidic chip will provide researchers many valuable opportunities to investigate the molecular nature of these cells and cancer metastasis and provide an opportunity to better understand the clinical importance of heterogeneous CTCs as biomarkers and therapeutic targets.

## MATERIALS AND METHODS

### Reagents

Anti-CD45, anti-EpCAM, anti-fibronectin, anti-ALDH1A1, anti-CD133, anti-vimentin, and anti-cytokeratin antibodies were from Abcam. Anti-snail, anti-Oct-4A, and anti-CD44 antibodies were from Cell Signaling. Anti-E-cadherin, anti-N-cadherin, anti-CD24, anti-CD44, anti-CD45, anti-cytokeratin, and anti-tubulin antibodies were from BD Transduction Laboratories.

### Cell culture

MCF-7 cells were obtained from the American Type Culture Collection (ATCC) and maintained in DMEM medium with 10% fetal bovine serum. For the sphere culture, single-cell suspensions of MCF-7 cells were suspended at a density of 5 x10^5^ cells/mL in DMEM/F12 containing 1x B27 supplement (Invitrogen), 20 ng/mL basic fibroblast growth factor (R&D), 20 ng/mL recombinant epidermal growth factor (Gibco), 100 U/mL penicillin and 100 μg/mL streptomycin and seeded into ultralow adherence dishes. Cultures were fed twice a week and maintained by weekly trypsinization and dissociation with a 23-gauge needle. After checking for single cells, cells were pelleted and suspended in mammosphere media at 5 x10^5^ cells/mL into an ultralow adherence dish [13].

### Western blot analysis

Cells were lysed in M2 buffer [13]. Equal amounts of cell extracts were resolved by 12% SDS-PAGE and analyzed by Western blot and visualized by enhanced chemiluminescence (ECL, Amersham).

### Reverse transcription-PCR

Total RNA was extracted from tumor specimens using the Mirvana RNA isolation kit (Ambion, Inc.) according to the manufacturer's instructions. PCR amplification was performed using the following primers: human snail gene sense (5′-CCTCsCCTGTCAGATGAGGAC-3′), snail gene antisense (5′-CCAGGCTGAGGTATTCCTTG-3′), twist gene sense (5′-GGAGTCCGCAGTCTTACGAG-3′), twist gene antisense (5′-TCTGGAGGACCTGG TAGAGG-3′), fibronectin gene sense (5′-CAGTG GGAGACCTCsGAGAAG-3′), fibronectin gene antisense (5′-TCCCTCsGGAACATCAGAAAC-3′), EpCAM gene sense (5′-GCGTTCGGGCTTCTGCTTGC-3′), EpCAM gene antisense (5′-CCGCTCsTCATCGCAGTCAGGA-3′) and actin gene sense (5′-CAGGTCATCACCATTGGCAATGAGC-3′), and actin gene antisense (5′-GATGTCCACGTCACACTTCATGA-3′). The final PCR products were resolved in 1.5% agarose gel and stained with ethidium bromide.

### Flow cytometry

Adherent and sphere cells were detached with accutase (Sigma) and incubated in staining buffer with anti-EpCAM (PE-conjugated), anti-CD44 (APC-conjugated) and anti-CD24 (FITC-conjugated) and finally analyzed by flow cytometry.

### Blood sample processing

Human blood samples were obtained from Severance Hospital (Seoul, Korea). We collected 7.5 mL peripheral blood in heparinized EDTA tubes (BD Vacutainer; Becton Dickinson, Heidelberg, Germany) from 32 patients with metastatic breast cancer and 5 healthy donors as a negative control. the samples were processed within six hours after collection. This study was approved by the Institutional Review Board (IRB) of Severance Hospital and all enrolled patients gave their informed consent. The clinical sample collection was carried out in accordance with the guidelines and protocols approved by the IRB of Severance Hospital.

### Identification and enumeration of CTCs and WBCs

An Olympus IX81-ZDC inverted microscope with a motorized stage was used to image the active area in a microfluidic filter chip. After completing the blood filtration step, when the blood flow stopped, the MOFF chip was washed with PBS to remove residual blood cells and the staining process was conducted in sequential steps of fixation (4% paraformaldehyde for 20 min), permeabilization (0.01% Triton X-100 reagent for 10 min) and cell staining [mixture of 4', 6-diamidino-2-phenylindole (DAPI), anti-cytokeratin PE (CK; CAM 5.2, BD Biosciences, CA) and CD45 FITC (BD Biosciences, CA) for 1 hr]. Captured images were carefully examined and the cells that stained positive for cytokeratin and negative for CD45 were scored as CTCs with consideration of the phenotypic morphological characteristics.

### Cell mass analysis using quantitative phase microscope

We used an interferometric microscope based upon Mach-Zehnder interferometry with a He-Ne laser (633 nm wavelength) as an illumination source [16]. This microscope measures the phase delay of the illumination light wave induced by cells relative to their surrounding medium, and the integrated phase delay is proportional to the mass of a cell excluding the aqueous content within the cell. The output beam from the laser was split into two, and one of the beams, called the sample beam, was sent through the cells on the sample stage and the transmitted wave was delivered to the camera with a magnification of 100x. The other beam was sent through free space and then interfered with the sample beam at the camera. From the interference image, the map of phase delay induced by the cells was obtained. The integral phase for each cell was converted into the nonaqueous mass of the cell [25].

## SUPPLEMENTARY FIGURES


