# Supplementary material for: Epithelial-to-mesenchymal transition leads to loss of EpCAM and different physical properties in circulating tumor cells from metastatic breast cancer
Source: Oncotarget. 2016 Mar 22;7(17):24677–87. doi: 10.18632/oncotarget.8250 (PMC5029733; doi:10.18632/oncotarget.8250)
Supplement: Supplementary file 1 [file oncotarget-07-24677-s001.pdf]

Epithelial-to-mesenchymal transition leads to loss of EpCAM and different physical properties in circulating tumor cells from metastatic breast cancer

SUPPLEMENTARY FIGURES

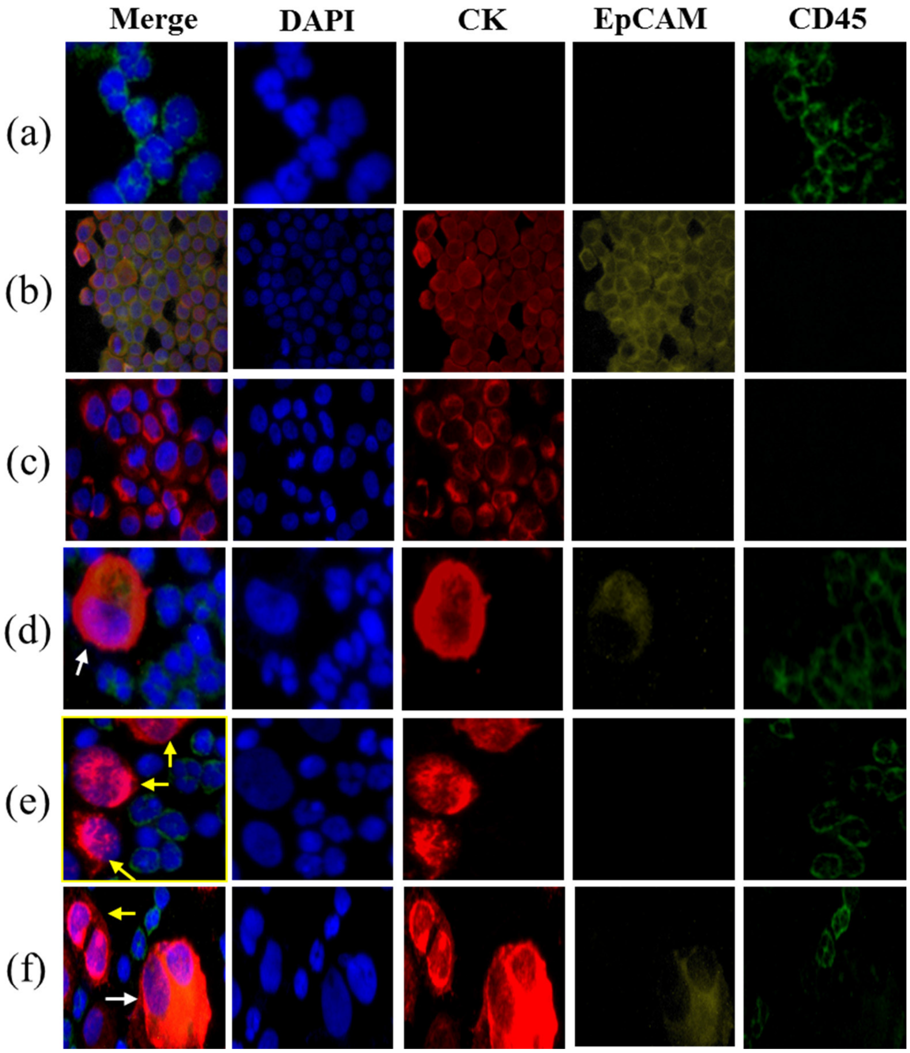

Supplementary Figure S1: Morphological analysis. a. White blood cells and **(b, c)** breast cancer cell lines were stained with immunofluorescence. **a.** White blood cells refer to DAPI+/CK-/EpCAM-/CD45+. **b.** EpCAM positive cell line MCF-7 cells refer to DAPI+/CK+/EpCAM+/CD45-. **c.** EpCAM negative cell line MDA-MB-231 cells refer to DAPI+/CK+/EpCAM-/CD45-. **d, e, f.** Breast cancer cell lines added to blood. White arrow indicates MCF-7 cells and yellow arrow indicates MDA-MB-231 cells.

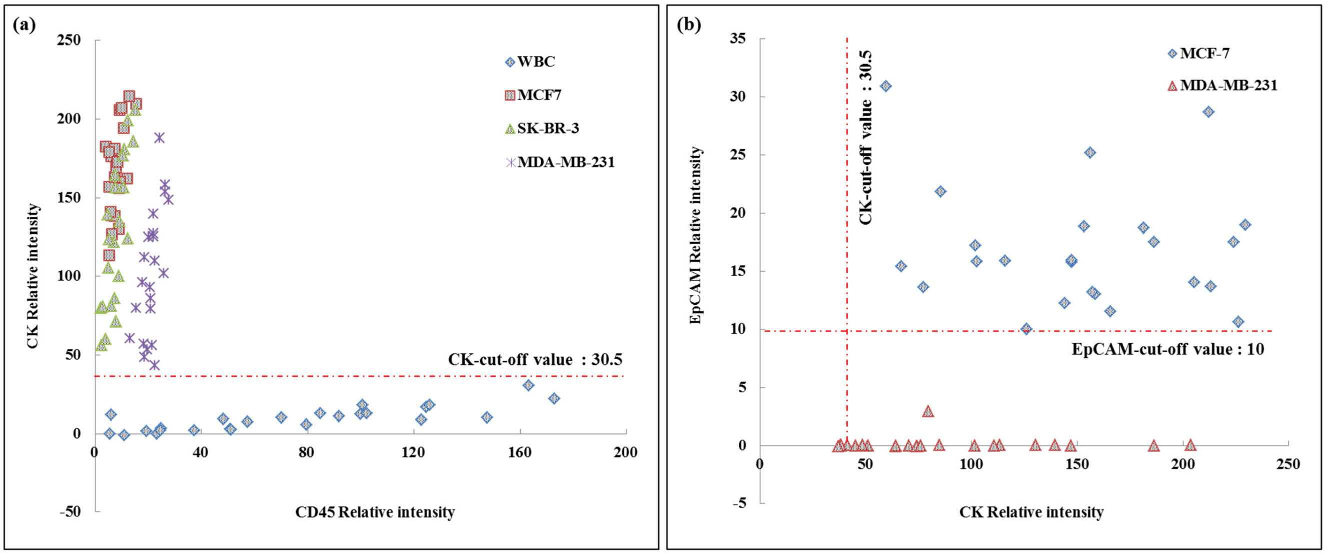

Supplementary Figure S2: Quantification of cytokeratin (CK) and EpCAM fluorescence intensity, according to cell type. a. The breast cancer cells (MCF-7, SK-BR-3, and MDA-MB-231) and WBCs are divided into cancerous or normal cell groups based on 30.5 CK relative intensity units. **b.** The MCF-7 cells and MDA-MB-231 cells are split into EpCAM positive and EpCAM negative cells based on 10 EpCAM relative intensity units.
